# Comparison of the functional and structural characteristics of rare *TSC2* variants with clinical and genetic findings

**DOI:** 10.1002/humu.23963

**Published:** 2019-12-19

**Authors:** Luiz G. Dufner Almeida, Santoesha Nanhoe, Andrea Zonta, Mitra Hosseinzadeh, Regina Kom‐Gortat, Peter Elfferich, Gerben Schaaf, Annegien Kenter, Daniel Kümmel, Nicola Migone, Sue Povey, Rosemary Ekong, Mark Nellist

**Affiliations:** ^1^ Department of Clinical Genetics Erasmus Medical Center Rotterdam The Netherlands; ^2^ Department of Genetics and Evolutionary Biology, Institute of Biosciences University of Sao Paulo Sao Paulo Brazil; ^3^ Department of Medical Sciences University of Turin Turin Italy; ^4^ Department of Developmental Biology Erasmus Medical Center Rotterdam The Netherlands; ^5^ Biochemistry and Structural Biology Section, Institute of Biochemistry University of Munster Munster Germany; ^6^ Department of Genetics, Evolution and Environment University College London London UK

**Keywords:** CRISPR/Cas9, functional assay, TORC1, *TSC2*, tuberous sclerosis complex, VUS

## Abstract

The *TSC1* and *TSC2* gene products interact to form the tuberous sclerosis complex (TSC), an important negative regulator of the mechanistic target of rapamycin complex 1 (TORC1). Inactivating mutations in *TSC1* or *TSC2* cause TSC, and the identification of a pathogenic *TSC1* or *TSC2* variant helps establish a diagnosis of TSC. However, it is not always clear whether *TSC1* and *TSC2* variants are inactivating. To determine whether *TSC1* and *TSC2* variants of uncertain clinical significance affect TSC complex function and cause TSC, in vitro assays of TORC1 activity can be employed. Here we combine genetic, functional, and structural approaches to try and classify a series of 15 *TSC2* VUS. We investigated the effects of the variants on the formation of the TSC complex, on TORC1 activity and on *TSC2* pre‐mRNA splicing. In 13 cases (87%), the functional data supported the hypothesis that the identified *TSC2* variant caused TSC. Our results illustrate the benefits and limitations of functional testing for TSC.

## 1 INTRODUCTION

Tuberous sclerosis complex (TSC) is an autosomal dominant disorder characterized by the development of hamartomas in a variety of organs and tissues, including the brain, skin, and kidneys. TSC affects approximately 1 in 10,000 individuals and is caused by inactivating mutations in either *TSC1* on chromosome 9q34 (MIM# 605284; van Slegtenhorst et al., 1997), or *TSC2* on chromosome 16p13.3 (MIM# 191092; European Chromosome 16 Tuberous Sclerosis Consortium, 1993). The identification of an inactivating *TSC1* or *TSC2* variant is sufficient for a diagnosis of TSC, even in the absence of clinical symptoms (Northrup & Krueger, 2013).

The *TSC1* and *TSC2* gene products, TSC1 (NP_000359.1) and TSC2 (NP_000539.2), interact to form a large GTPase activating protein (GAP) complex (Hoogeveen‐Westerveld et al., 2012; Menon et al., 2014) that acts on the GTPase RAS homolog expressed in brain (RHEB) to inhibit RHEB–GTP‐dependent activation of the mechanistic target of rapamycin complex 1 (TORC1; Huang & Manning, 2008). Loss or inactivation of the TSC complex results in constitutive TORC1 activity and increased phosphorylation of the downstream targets of TORC1, including p70 S6 kinase (S6K), ribosomal protein S6, and 4E‐BP1 (Huang & Manning, 2008).

The *TSC1* and *TSC2* Leiden Open Variant Databases (LOVD; http://www.lovd.nl/TSC1 and http://www.lovd.nl/TSC2) currently list >4,000 variants identified in individuals with TSC, including >1,000 variants in *TSC1* and >3,000 in *TSC2*. The majority of these variants are clearly inactivating, resulting in the truncation of the *TSC1* or *TSC2* open reading frame (ORF). However, for a significant minority of variants (currently >500), it is not yet clear whether they are disease‐causing. According to the guidelines of the American College of Medical Genetics and Genomics, in vitro functional assays can help establish or exclude pathogenicity for variants of uncertain clinical significance (VUS; Ghosh, Harrison, Rehm, Plon, & Biesecker, 2018; Richards et al., 2015), and assessment of the effects of *TSC1* and *TSC2* variants on TSC complex activity has been used to establish pathogenicity (Dunlop et al., 2011; Hoogeveen‐Westerveld et al., 2011; Northrup & Krueger, 2013; W. Qin et al., 2010). Here we apply new functional and structural approaches to investigate the likely pathogenicity of 15 novel *TSC2* VUS identified in individuals suspected of TSC and submitted to the *TSC2* LOVD.

## 2 MATERIALS AND METHODS

### 2.1 Generation of *TSC1* and *TSC2* knockout cells using CRISPR/Cas9 genome editing

Guide sequences targeting *TSC2* exon 2 (5′‐caccgacggagtttatcatcaccg‐3′ and 5′‐aaaccggtgatgataaactccgtc‐3′) and intron 38 (5′‐caccggttatcgccacgcaccact‐3′ and 5′‐aaacagtggtgcgtggcgataacc‐3′) and *TSC1* exon 3 (5′‐caccgtgggccattctctcgctcga‐3′ and 5′‐aaactcgagcgagagaatggcccac‐3′) and exon 23 (5′‐cacccagtcggtgggagacgacta‐3′ and 5′‐aaactagtcgtctcccaccgactg‐3′) were selected using online CRISPR design tools (Cong et al., 2013, Stemmer et al., 2015), cloned into the pX458 and pX459 vectors (Ran, Hsu, & Wright, 2013) and transfected into human embryonic kidney (HEK) 293T cells, following a previously described protocol (Chantranupong et al., 2014). Cells were cultured in six‐well plates in Dulbecco's modified Eagle's medium (DMEM; Lonza, Verviers, Belgium) supplemented with 10% fetal bovine serum, 50 U/ml penicillin and 50 μg/ml streptomycin (DMEM++), in a 5% carbon dioxide humidified incubator, until 50–70% confluent and then transfected using lipofectamine 2000 (Invitrogen, Carlsbad, CA). Twenty‐four hours later the cells were trypsinized, pooled, and transferred to a single 10‐cm plate. Samples were retained for the analysis of GFP expression and for DNA isolation. Six hours after reseeding, 3 μg/ml puromycin was added to the culture medium for 48 hr. After a 24 hr recovery phase in DMEM++, cells expressing GFP were single‐cell sorted using a FACSARIA III (BD Biosciences, San Jose, CA) into 96‐well plates precoated with poly‐d‐lysine. The sorted cells were cultured until colonies became apparent and could be trypsinized, expanded, and tested for target gene inactivation, and activation of TORC1 signaling.

### 2.2 In vitro functional assessment of the effects of *TSC2* variants on TSC complex activity

Expression constructs encoding the *TSC2* variants were derived by site‐directed mutagenesis. In each case, the complete ORF of the mutated construct was verified by sequence analysis. In contrast to previous studies (Hoogeveen‐Westerveld et al., 2013; Hoogeveen‐Westerveld et al., 2011), the wild‐type *TSC2* expression construct included codons 1272–1295, corresponding to the alternatively spliced exon 32 of *TSC2* (NM_000548.3, GI:116256351; Ekong et al., 2016). Other constructs have been described previously (Hoogeveen‐Westerveld et al., 2011). Both the wild‐type *TSC1* (NM_000368.4; GI: 2331280; van Slegtenhorst et al., 1998) and *S6K* (2B4) expression constructs encode C‐terminal myc‐epitope tags. Variants were compared with wild‐type *TSC2* (NM_000548.3, GI:116256351; referred to as TSC2) and to the inactive *TSC2* c.1832G>A, p.(R611Q) variant (referred to as R611Q), one of the most frequently detected causative *TSC2* variants for TSC (http://www.lovd.nl/TSC2).

Antibodies were purchased from Cell Signaling Technology (Danvers; 1A5, anti‐T389 phospho‐S6K mouse monoclonal; anti‐myc tag rabbit polyclonal; 9B11, anti‐myc tag mouse monoclonal; 91B2, anti‐S235 phospho‐S6 rabbit monoclonal; and 5G10, anti‐total S6 rabbit monoclonal), Merck (Haarlem, The Netherlands; MAB374, anti‐glyceraldehyde phosphate dehydrogenase mouse monoclonal) or Li‐Cor Biosciences (Lincoln; goat anti‐rabbit 680 nm and goat anti‐mouse 800 nm conjugates). Rabbit polyclonal antibodies raised against human TSC2 (1895) and TSC1 (2197) have been described previously (van Slegtenhorst et al., 1998).

The transfection‐based immunoblot assay for functional assessment of *TSC2* variants was performed as described previously (Hoogeveen‐Westerveld et al., 2011), except that the variants were expressed in the *TSC2* knockout (KO) and *TSC1:TSC2* double knockout (DKO) HEK 293T subclones. First, 1–2 × 10^5^ cells were seeded per well into 24‐well culture dishes. The following day the cells were transfected with 0.8 µg DNA (1:2:1 ratio *TSC2*:*TSC1*:*S6K*) and 4 µg polyethyleneimine and harvested 24 hr later in lysis buffer (50 mM Tris‐HCl, pH 7.6, 100 mM NaCl, 50 mM NaF, and 1% Triton X‐100 containing the Complete^TM^ protease inhibitor cocktail [Roche Molecular Biochemicals, Woerden, The Netherlands]). After centrifugation (10,000*g*, 10 min, 4°C), the supernatant fractions were subjected to immunoblotting.

TSC complex formation was assessed by coimmunoprecipitation. Briefly, 1.5 × 10^6^ *TSC1:TSC2* DKO HEK 293T cells were seeded into 6‐cm dishes and transfected with 10 µg polyethyleneimine and 2 µg of a 1:1 mix of expression constructs encoding wild‐type myc‐tagged TSC1 and either wild‐type TSC2, one of the *TSC2* variants, or the pcDNA3 expression vector. Twenty‐four hours after transfection, the supernatant fractions were divided between (a) EZ Red anti‐myc affinity beads (Sigma‐Aldrich, Zwijndrecht, The Netherlands) for immunoprecipitation of exogenous myc‐tagged TSC1, and (b) a rabbit polyclonal anti‐TSC2 antiserum (van Slegtenhorst et al., 1998), followed by Protein A‐agarose beads (GE Healthcare, Eindhoven, The Netherlands), for immunoprecipitation of the exogenous TSC2 variant proteins. After 3–4 hr gentle rotation at 4°C, the beads were recovered by centrifugation (1,000*g*, 15 s) and washed three times with a >20‐fold excess of lysis buffer before immunoblot analysis.

### 2.3 Structural assessment of the *TSC2* variants

Secondary structure predictions were made using PSIPRED (Buchan, Minneci, Nugent, Bryson, & Jones, 2013). The X‐ray crystal structures of the N‐terminal HEAT repeat domain of TSC2 from the fungus *Chaetomium thermophilum* (Zech, Kiontke, Mueller, Oeckinghaus, & Kummel, 2016) and the catalytic domain of RapGAP (Daumke, Weyland, Chakrabarti, Vetter, & Wittinghofer, 2004; Scrima et al., 2008), which shares ~25% sequence identity with the GAP domain of TSC2, were used to model the human structure with SWISS‐MODEL (Arnold, Bordoli, Kopp, & Schwede, 2006). Structural predictions were made independently of the functional test results.

### 2.4 In vitro assessment of the effects of *TSC2* variants on *TSC2* pre‐mRNA splicing

*TSC2* coding exons and the surrounding intronic sequences were amplified by polymerase chain reaction (PCR) and cloned into the pSPL3 splicing vector (Halim et al., 2017; Van Der Werf et al., 2012). *TSC2* VUS were introduced by site‐directed mutagenesis and all constructs were verified by sequence analysis (Table 1). Between 3–5 × 10^5^ HEK 293T cells were seeded per well into six‐well culture dishes and transfected the following day with 8 µg polyethyleneimine and 1.6 µg of the wild‐type or variant splicing constructs. RNA was isolated 24 hr after transfection using the RNeasy mini kit (Qiagen, Venlo, The Netherlands). Reverse transcriptase (RT) PCR was performed using the iScript complementary DNA synthesis kit (Bio‐Rad, Hercules) and primers specific for pSPL3‐derived transcripts: SD6_f 5′‐tctgagtcacctggacaacc‐3′ and SA2_r 5′‐gctcacaaataccactgagat‐3′. PCR products were analyzed by agarose gel electrophoresis and Sanger sequencing.

**Table 1:** Functional assessment of *TSC2* variants

Group	cDNA	Splicing assay	Structural classification	Functional assessment	ACMG evidence	
					Pathogenic	Benign
1	c.250G>A p.(A84T)	Only canonical splice product detected	Surface; tolerated	No evidence for an effect on function	PM2	BS3
1	c.4225C>G p.(R1409G)	Only canonical splice product detected	Disordered region; tolerated	No evidence for an effect on function	PM2	BS3
1	c.4966G>A p.(D1656N)	Only canonical splice product detected	Surface; tolerated	No evidence for an effect on function		BS3
2	c.4966G>T p.(D1656Y)	r.4960_4989del p.(E1655Pfs*40)	Surface; tolerated	No evidence for effect on function	PS3, PS4, PM2, PS2, PP1	
2	c.1477C>G p.(L493V)	r.1444_1477delinsG p.(482_493delinsV)	No prediction	p.L493V: possible disruption of TSC1–TSC2 interaction	PM2, PM5, PS3	
				p.482_493delinsV: inactivates TSC complex		
2	c.440C>A p.(T147K)	Not done	Packing defect	Disrupts TSC complex function	PM2, PS3	
2	c.839T>C p.(M280T)	Not done	Packing defect	Disrupts TSC complex function	PP1, PS3	
2	c.922C>T p.(R308W)	Not done	Interferes with TSC1 binding	Disrupts TSC complex function	PS3	
2	c.1106T>C p.(L369P)	Not done	Helix breaking	Disrupts TSC complex function	PM2, PS3	
2	c.1343T>G p.(L448R)	Not done	No prediction	Disrupts TSC complex function	PM2, PM5, PS3	
2	c.1511T>A p.(V504D)	Not done	No prediction	Disrupts TSC complex function	PS3	
2	c.1574_1576del p.(525del)	Not done	No prediction	Disrupts TSC complex function	PM4, PM5, PS3	
2	c.3134C>T (p.S1045F)	r.3132_3284del p.(1044_1094del)	No prediction	p.S1045F: disrupts TSC complex function	PP1, PS3	
				p.1044_1094del: inactivates TSC complex		
2	c.3589A>T (p.I1197F)	Not done	No prediction	Disrupts TSC complex function	PS3	
3	c.2747T>C p.(L916P)	Not done	No prediction	Inactivates TSC complex	PS3	
3	c.4709_4714del p.(1570_1571del)	Not done	Packing defect	Inactivates TSC complex	PM4, PS3	

*Note*: Variants are listed according to the results of the functional assessment and structural predictions. Evidence of pathogenicity and evidence of benign impact, according to the guidelines of the ACMG (Richards et al., 2015), are indicated. Variants in Group 1 did not affect TSC complex formation, activity or splicing; variants in Group 2 disrupted TSC complex function and/or *TSC2* pre‐mRNA splicing; variants in Group 3 completely inactivated TSC complex‐dependent suppression of TORC1 activity. Nucleotide numbering is according to the *TSC2* reference transcript NM_000548.3.

Abbreviations: ACMG, American College of Medical Genetics and Genomics; cDNA, complementary DNA; TORC1, target of rapamycin complex 1.

## 3 RESULTS

### 3.1 Characterization of *TSC1* and *TSC2* KO and DKO cells

**Figure 1:**
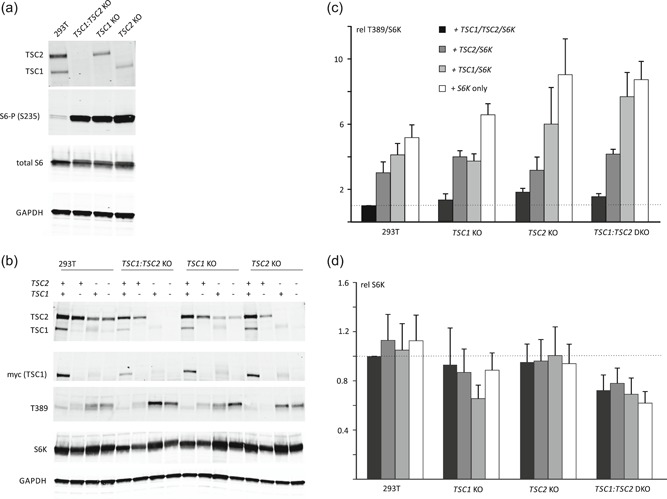
TSC1 and TSC2 knockout (KO) clone analysis. CRISPR/Cas9 genome editing was used to inactivate *TSC2* and/or *TSC1* in HEK 293T cells. The *TSC1* knockout (*TSC1* KO), *TSC2* knockout (*TSC2* KO), and *TSC1:TSC2* double knockout (*TSC1:TSC2* DKO) subclones were compared to the parental HEK 293T cell‐line (293T). To characterize TORC1 signaling in the different cell‐lines, cells were starved of growth factors (serum) overnight before harvesting. The cleared cell lysates were analyzed by immunoblotting. (a) TSC1 and/or TSC2 protein signals were absent from the *TSC1* and/or *TSC2* KO cells and, in contrast to the parental 293T cells, the KO cells showed robust S6‐S235 phosphorylation (S6‐P (S235)), a marker for TORC1 activity. Total protein content of the lysates was estimated from the total S6 and GAPDH signals. To investigate the effects of exogenous *TSC1* and *TSC2* expression the KO cells were transfected with *S6K*, *TSC1*, and/or *TSC2* expression constructs, as indicated, and analyzed by immunoblotting. (b) The total S6K (S6K) and T389‐phosphorylated S6K (T389) signals were determined per cell‐line in three independent experiments, and the mean T389/S6K ratios (c) and mean total S6K signals (d), relative to 293T cells expressing exogenous TSC complexes, were determined. An increased T389/S6K ratio corresponds to increased TORC1 activity. The T389/S6K ratio was highest in the *TSC2* KO and *TSC1:TSC2* DKO cells expressing S6K only and was reduced by expression of either *TSC2* or *TSC1* and *TSC2*. Error bars represent the standard error of the mean. GAPDH, glyceraldehyde phosphate dehydrogenase; HEK, human embryonic kidney; TORC1, target of rapamycin complex 1

Previously, we assessed the effect of *TSC2* variants on TSC complex activity by expressing the variants in HEK 293T cells (Hoogeveen‐Westerveld et al., 2013; Hoogeveen‐Westerveld et al., 2011). HEK 293T cells express endogenous, active TSC complexes, resulting in background signals, and reduced TORC1 activity. Furthermore, interactions between the exogenous variant and endogenous (wild‐type) TSC complexes might influence variant activity. To avoid these problems, we used CRISPR/Cas9 genome editing to delete *TSC1* and/or *TSC2* from our HEK 293T cells. Breakpoint PCR and Sanger sequencing confirmed that the CRISPR/Cas9 targeting was successful in multiple subclones. We verified that the KO clones did not express TSC2 and/or TSC1 and that TORC1 signaling was constitutively activated (Figure 1a). Next, we compared the effects of exogenous expression of wild‐type *TSC2* and *TSC1* in the KO and DKO subclones with the original HEK 293T cells. TORC1 activity in the KO and DKO cells, as estimated from the S6K‐T389 phosphorylation status (T389/S6K ratio; see below), was sensitive to exogenous expression of TSC complex components (Figure 1b,c). Furthermore, consistent with previous results (Nellist et al., 1999), the expression of TSC2 was required to ensure stable expression of TSC1 in the cytosolic fraction (Figure 1b).

### 3.2 Functional assessment of *TSC2* variants

*TSC2* variants were identified during the diagnostic screening of a series of individuals suspected of TSC and were submitted to the *TSC2* LOVD (http://www.lovd.nl/TSC2). At submission, there was no detailed clinical information available and none of the variants were present in the ExAC and EVS population datasets, making it difficult to assess pathogenicity. Therefore the variants were classified as VUS and considered for in vitro functional analysis. The variants are listed in Table 2, along with the additional clinical and genetic data that we were subsequently able to obtain on the individuals carrying the variants. The available pedigree information for five families segregating *TSC2* VUS is shown in Figure S1. All variants were assessed using ALAMUT variant prediction software (Interactive Biosoftware, France). Nucleotide numbering is according to the human reference transcript NM_000548.3, with +1 corresponding to the A of the ATG translation initiation codon in the reference sequence (http://www.hgvs.org/mutnomen). The initiation codon is codon 1.

**Table 2:** *TSC2* VUS identified in individuals suspected of TSC

cDNA	Phenotypic features of individuals with the variant
c.250G>A p.(A84T)	Cognitive disability and single HM
c.4225C>G p.(R1409G)	HM, FA, UF, retinal hamartoma, and renal AML
c.4966G>T p.(D1656Y)	1.4: AF; 1.3: epilepsy, abnormal brain CT, FA, UF, renal AML, LAM; 1.2: FA, UF (Figure S1a)
c.1477C>G p.(L493V)	2.1: Cortical dysplasia, SEN, HM, epilepsy, behavioral problems; 2.2: epilepsy, cortical dysplasia
c.440C>A p.(T147K)	Multiple HM; brain MRI, cardiac US, abdominal US, and eye exams all normal
c.839T>C p.(M280T)	3.3: Epilepsy, CR, cortical dysplasia; 3.2: HM, renal US abnormality
	4.1: CR, SEN; 4.2: epilepsy, cortical dysplasia, HM; 4.3: epilepsy, HM, renal cell carcinoma; 4.4: HM; 4.5: normal skin and neurological exam (Figure S1b)
c.922C>T p.(R308W)	5.3: CR; 5.1: HM, cortical dysplasia
	6.1: Epilepsy, cortical dysplasia, SEN, HM; 6.2: cortical dysplasia
c.1106T>C p.(L369P)	7.3: Cortical dysplasia, SEN, epilepsy, cognitive disability; 7.2: cortical dysplasia, renal cyst
c.1343T>G p.(L448R)	CR, HM, and unspecified brain MRI abnormalities
c.1511T>A p.(V504D)	8.3: CR, Wolff–Parkinson–White syndrome; 8.1: brain MRI, renal and cardiac US, skin and eye exams all normal (Figure S1c)
	9.3: Epilepsy, cognitive disability, cortical dysplasia; 9.2: brain CT, renal US, and eye exams all normal
c.1574_1576del p.(525del)	Epilepsy, SEN, and cortical dysplasia
c.3134C>T (p.S1045F)	10.4: Epilepsy, cortical dysplasia, SEN, HM, AML; 10.3: epilepsy, FA, UF, cortical dysplasia; 10.2: FA, UF (Figure S1d)
c.3589A>T (p.I1197F)	11.3: HM, FA, CR, SEN, renal AML, retinal hamartoma, epilepsy, cognitive disability; 11.1: cardiac and renal US, brain MRI, skin and eye exams all normal
c.2747T>C p.(L916P)	CR, cortical dysplasia, SEN, and epilepsy
c.4709_4714del p.(1570_1571del) (1570del2)	12.3: FA, HM, cortical dysplasia, epilepsy, cognitive disability; 12.4: FA, HM, UF, shagreen patch, cortical dysplasia, West syndrome/infantile spasms, cognitive disability

*Note*: Nucleotide numbering is according to the *TSC2* reference transcript NM_000548.3.

Related individuals are indicated according to family number (e.g., family 1: individual 1.1, individual 1.2).

Abbreviations: AML, angiomyolipoma; cDNA, complementary DNA; CR, cardiac rhabdomyoma; CT, computed tomography; FA, facial angiofibroma; HM, hypomelanotic macule; LAM, lymphangioleiomyomatosis; MRI, magnetic resonance imaging; SEN, subependymal nodule; TSC, tuberous sclerosis complex; UF, ungual fibroma; US, ultrasound; VUS, variants of uncertain clinical significance.

In total, we derived expression constructs encoding 18 different *TSC2* variants (Table 1). Sequencing of the entire ORF of the mutated constructs confirmed the presence of the intended nucleotide changes and identified a single, additional synonymous change, c.1473G>A (p.S491S), in the expression construct encoding the *TSC2* c.4966G>T (p.D1656Y) variant.

To assess the effects of the *TSC2* variants on TORC1 activity, S6K‐T389 phosphorylation in cells expressing the variants together with myc‐tagged wild‐type TSC1 and S6K was estimated by immunoblotting (Figure 2a). Mean values for the TSC2 (Figure 2b), TSC1 (Figure 2c), and S6K (Figure 2e) signals, and the mean ratios of T389‐phosphorylated S6K to total S6K (T389/S6K ratio; Figure 2d) were determined for all the variants tested, relative to the values for wild‐type TSC2.

Figure 2Functional assessment of the *TSC2* variants. The signals for TSC2, TSC1, total S6K (S6K), and T389‐phosphorylated S6K (T389) were determined per variant, relative to the wild‐type control (TSC2) in at least four independent transfection experiments. An example of an immunoblot is shown in (a). The mean TSC2 (b), TSC1 (c), and S6K (e) signals and mean T389/S6K ratio (d) are shown for each variant. In each case, the dotted line indicates the signal/ratio for wild‐type TSC2 (=1.0). Error bars represent the standard error of the mean. Mean signals for the p.L448R and p.482_493delinsV (482del11) variants are shown separately as they were not included on the blot shown in (a). Amino acid changes are given according to the *TSC2* Leiden Open Variation Database (*TSC2* reference transcript sequence NM_000548.3; http://www.lovd.nl/TSC2; see main text for variant abbreviations). Variants classified in Group 1 (no effect on TSC complex function) are shown in black, Group 2 variants (disrupt TSC complex activity) are in gray, and Group 3 variants (inactivate TSC complex) are in white (see main text for details). (b) Mean signals for the TSC2 variants, relative to wild‐type TSC1–TSC2 (TSC2; TSC2 signal = 1). Values that were significantly different from the wild‐type control (TSC2) are indicated with an asterisk (*p* < .025; Student's *t* test). (c) Mean TSC1 signals in the presence of the TSC2 variants, relative to wild‐type TSC1–TSC2 (TSC2; TSC1 signal = 1). Values that were significantly different from the wild‐type control (TSC2) are indicated with an asterisk (*p* < .025; Student's *t* test). (d) Mean T389/S6K ratios for the TSC2 variants, relative to wild‐type TSC1–TSC2 (TSC2; T389/S6K ratio = 1). Ratios that were significantly increased compared to the wild‐type control (TSC2) are indicated with an asterisk (*p* < .025; Student's *t* test). (e) Mean total S6K signals in the presence of the TSC2 variants, relative to wild‐type TSC2 (TSC2; S6K signal = 1). Values that were significantly different from the wild‐type control (TSC2) are indicated with an asterisk (*p* < .025; Student's *t* test)

**Figure:**
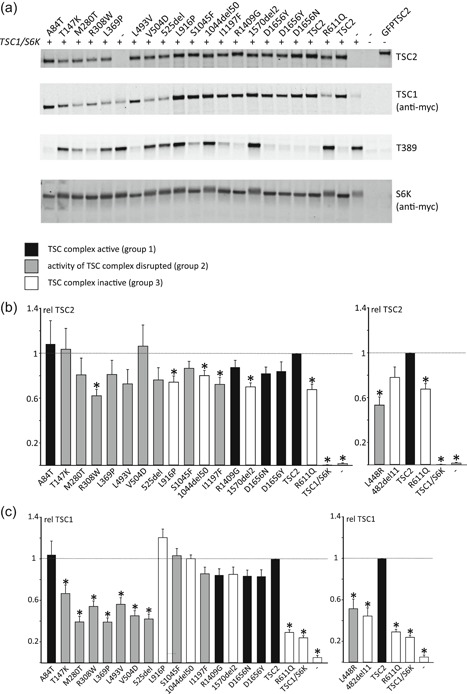

**Figure:**
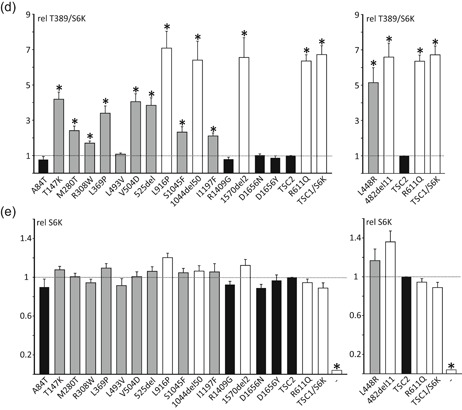

To investigate their effects on TSC complex formation we coexpressed the TSC2 variants with myc‐tagged wild‐type TSC1 in the *TSC1:TSC2* DKO HEK 293T cells and isolated TSC complexes by immunoprecipitation using antibodies specific for either the TSC2 (Figure 3a) or TSC1 (Figure 3d) subunit. The mean signals for immunoprecipitated and coimmunoprecipitated TSC1 and TSC2 were determined for all the variants tested, relative to the values for the wild‐type TSC complex. All the *TSC2* variants could be immunoprecipitated effectively (Figure 3b), and the signals for coimmunoprecipitated TSC1 (Figure 3c) were used to estimate the ability of the *TSC2* variant to form a stable TSC complex with TSC1. Reduced coimmunoprecipitation of TSC2 and TSC1, using antibodies specific for the TSC1 subunit (Figure 3d–f), confirmed that specific *TSC2* variants had a reduced ability to form stable TSC complexes.

Figure 3Immunoprecipitation of variant TSC complexes. *TSC1:TSC2* double knockout cells were cotransfected with expression constructs encoding C‐terminal myc‐tagged TSC1 and either TSC2 or a TSC2 variant. TSC complexes were immunoprecipitated from the cleared cell lysates using either antibodies specific for the (TSC1) myc‐epitope tag (anti‐myc IP) or for an epitope close to the TSC2 C‐terminus (TSC2 IP). Immunoprecipitated TSC complexes were detected by immunoblotting (a and d) and the TSC2 and TSC1 signals were determined per variant relative to the wild‐type control (TSC2) in two independent transfection experiments. (b) The mean signals are shown for immunoprecipitated TSC2 (TSC2 IP) and (c) coimmunoprecipitated TSC1 (TSC1 coIP (TSC2 IP), (e) immunoprecipitated TSC1 (TSC1 IP), and (f) coimmunoprecipitated TSC2 (TSC2 co IP (TSC1 IP). In each case, the dotted line indicates the signal corresponding to wild‐type TSC2 (=1.0). Error bars represent the standard error of the mean. Mean signals for the p.482_493delinsV (482del11) variant are shown separately as it was not included on the blots shown in (a) and (d). Amino acid changes are given according to the *TSC2* Leiden Open Variation Database (*TSC2* reference transcript sequence NM_000548.3; http://www.lovd.nl/TSC2; see main text for variant abbreviations). Variants classified in Group 1 (no effect on TSC complex function) are shown in black, Group 2 variants (disrupt TSC complex activity) are in gray and Group 3 variants (inactivate TSC complex) are in white (see main text for details)

**Figure:**
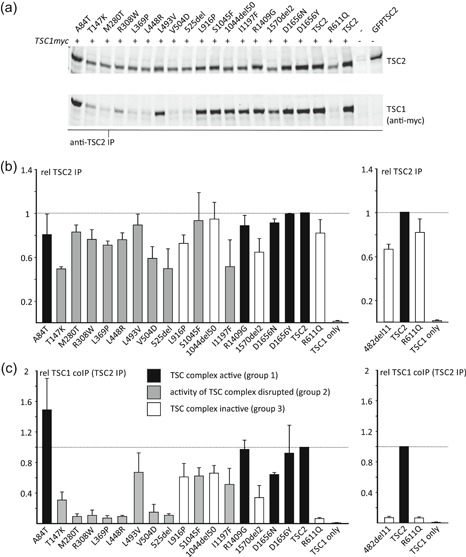

**Figure:**
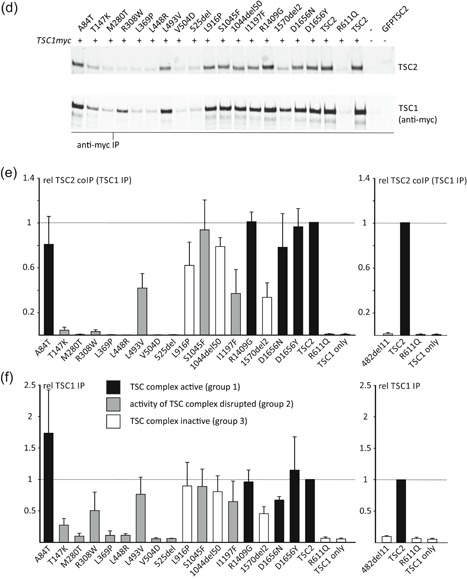

To assess the effects of *TSC2* variants on pre‐mRNA splicing we transfected HEK 293T cells with the corresponding wild‐type and variant splice assay constructs (Table 3). Canonical and noncanonical spliced mRNA molecules were amplified by RT‐PCR using the splice vector‐specific primers and analyzed by agarose gel electrophoresis followed by Sanger sequencing (see below).

**Table 3:** Overview of the in vitro splicing assay results

Variant	Splice construct(s) (exon)	r.	Conclusion
c.250G>A, p.(A84T)	c.226‐127_c.336+97 c.250G>A (exon 4)	r.250G>A	No evidence for an effect on splicing
c.1477C>G, p.(L493V)	c.1444‐146_c.1599+188 c.1477C>G (exon 15)	r.1444_1477delinsG, p.(482_493delinsV)	In‐frame deletion due to utilization of a noncanonical 3′acceptor site at c.1477 (see Figure S2a)
c.3134C>T, p.(S1045F)	c.2967‐73_c.3397+86 c.3134C>T (exons 27–29)	r.3132_3284del p.1044_1094del	In‐frame deletion due to skipping of exon 27 (see Figure S2d)
c.4225C>G, p.(R1409G)	c.4006‐101_c.4493+68 c.4225C>G (exon 34)	r.4225C>G	No evidence for an effect on splicing
c.4966G>T p.(D1656Y)	c.4850‐95_4989+113 c.4966G>T (exon 38)	r.4961_4989del, p.(E1655Pfs*40)	Frameshift due to utilization of a noncanonical 5′ donor site at c.4960 (see Figure S2c)
c.4966G>A p.(D1656N)	c.4850‐95_4989+113 c.4966G>A (exon 38)	r.4966G>A	No evidence for an effect on splicing (see Figure S2b)

*Note*: Nucleotide numbering is according to the *TSC2* reference transcript NM_000548.3.

We classified the *TSC2* variants into three groups (Table 1) according to the observed effects on (a) TSC complex‐dependent TORC1 activity, as estimated from the T389/S6K ratio (Figure 2b), (b) on the formation of TSC complexes, as estimated by coimmunoprecipitation (Figure 3), and (c) on the effect of the variant on *TSC2* pre‐mRNA splicing, as assessed in an in vitro splicing assay (Table 3). Variants in Group 1 did not affect TSC complex formation or activity and did not affect splicing. TORC1 activity in the presence of the Group 1 variants was significantly reduced compared with TORC1 activity in the absence of TSC2 (*p* < .025; paired Student's *t* test with Bonferroni correction) and was not significantly increased compared with wild‐type TSC2 (*p* > .025; paired Student's *t* test with Bonferroni correction). Variants in Group 2 disrupted TSC complex function but did not inactivate the complex completely and/or resulted in the production of abnormal splice products. We defined variants that disrupted TSC complex activity as those with significantly increased TORC1 activity compared with wild‐type TSC2 (*p* < .025; paired Student's *t*‐test with Bonferroni correction) and with significantly reduced TORC1 activity compared with the absence of TSC2 (*p* < .025; paired Student's *t* test with Bonferroni correction). Variants in Group 3 inactivated the TSC complex completely: TORC1 activity was significantly increased compared with wild‐type TSC2 (*p* < .025; paired Student's *t* test with Bonferroni correction) and was not significantly different to TORC1 activity in the absence of TSC2 (*p* > .025; paired Student's *t* test with Bonferroni correction).

#### 3.2.1 Group 1 variants

**Figure 4:**
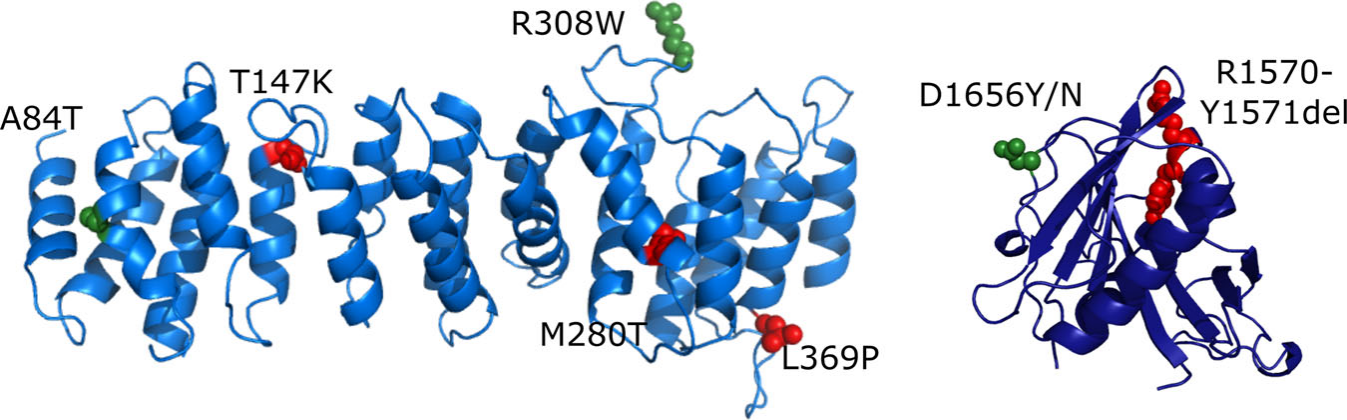
Structural predictions for *TSC2* variants. Models for the human TSC2 N‐terminal domain (left) and GTPase activating protein (GAP) domain (right) were generated with SWISS‐MODEL based on PDBID:5HIU (Zech et al., 2016) and PDBID:1SRQ (Daumke et al., 2004), respectively. Amino acid changes are given according to the *TSC2* Leiden Open Variation Database (*TSC2* reference transcript sequence NM_000548.3; http://www.lovd.nl/TSC2); changes to the residues shown in green are predicted to be structurally tolerated; changes to the residues shown in red are likely to disrupt protein structure

The *TSC2* c.250G>A p.(A84T) variant was identified in an individual with intellectual disability and a single hypomelanotic macule. Structural analysis indicated that the A84 residue is located on the surface of TSC2 (Figure 4) and that the A84T substitution is unlikely to affect protein folding or stability. Splice site prediction analysis did not suggest that the c.250G>A substitution would affect splicing and only the canonical exon 4 splice product was detected in the in vitro splice assay (Table 3).

The *TSC2* c.4225C>G p.(R1409G) variant was identified in an individual who fulfilled the clinical criteria for TSC. R1409 maps to a region of TSC2 (amino acids 1,207–1,479) that is predicted to be intrinsically disordered and it is unlikely that the R1409G substitution causes a structural defect. Only the canonical exon 34 splice product was detected in the in vitro splice assay (Table 3).

#### 3.2.2 Group 2 variants

**Figure 5:**
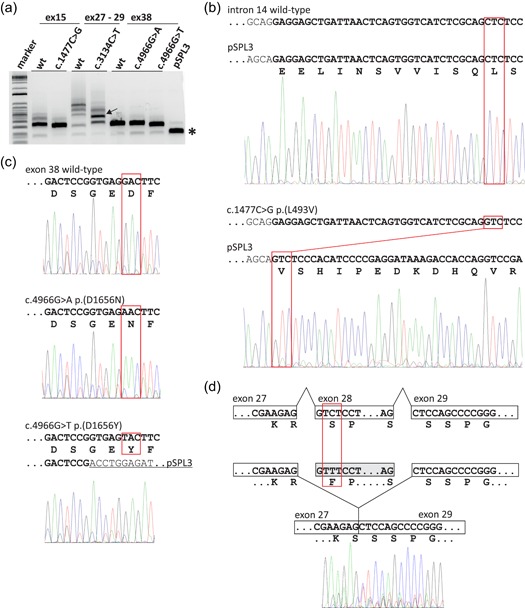
In vitro analysis of TSC2 pre‐mRNA splicing. RT‐PCR was performed on RNA isolated from cells transfected with constructs encoding either wild‐type (wt) or variant *TSC2* exons 15 (ex15), 27–29 (ex27–29) or 38 (ex38); the pSPL3 splicing vector without an insert was included as a control (pSPL3). (a) Agarose gel of the RT‐PCR amplification products. Sanger sequencing results are shown in (b–d). A constitutive ~230 bp splice product of the vector donor and acceptor sites is indicated with an asterisk and is the primary cause of the background peaks visible in the electropherograms shown in (b–d). Multiple RT‐PCR products were obtained from both the wild‐type and c.3134C>T p.(S1045F) variant constructs containing TSC2 exons 27–29. The arrow indicates the major product derived from the c.3134C>T p.(S1045F) variant construct. This product was excised from the gel before sequencing (see (d), below). (b) Noncanonical *TSC2* splicing caused by the c.1477C>G p.(L493V) substitution. Exonic DNA sequences are indicated in bold and the corresponding amino acid sequence is shown below the sequence of the pSPL3–*TSC2* exon 15 splice product; codon 493 is highlighted in red. The first 11 codons of exon 15 are missing from the sequence derived from the c.1477C>G p.(L493V) variant (lower panel). (c) Noncanonical *TSC2* splicing caused by the c.4966G>T p.(D1656Y) substitution (bottom panel), but not the c.4966G>A p.(D1656N) substitution (middle panel). Exonic sequences are indicated in bold; sequences corresponding to the pSPL3 acceptor site are underlined; codon 1656 is highlighted in red. The c.4966G>T p.(D1656Y) substitution results in the utilization of a noncanonical donor site at c.4960 (see Figure S2c). (d) *TSC2* exon 28 skipping associated with the c.3134C>T p.(S1045F) substitution. The sequence obtained from the RT‐PCR product indicated with an arrow in (a) is shown. Sequences corresponding to exon 28 were absent from the electropherogram. bp, base pair; mRNA, messenger RNA; RT‐PCR, reverse transcriptase‐polymerase chain reaction

The *TSC2* c.1477C>G, p.(L493V) variant was identified in a parent and child, both fulfilling the diagnostic criteria for TSC, and the same variant has been described in other individuals with TSC (Hoogeveen‐Westerveld et al., 2013, Langkau et al., 2002). The TSC2 p.L493V variant was associated with a significantly reduced TSC1 signal (Figure 2c), consistent with previous findings (Hoogeveen‐Westerveld et al., 2013) and suggestive of an effect on the TSC1–TSC2 interaction. Nonetheless, a robust interaction between TSC1 and the TSC2 p.L493V variant was detected by coimmunoprecipitation (Figure 3), and the variant was able to inhibit TORC1 activity as effectively as wild‐type TSC2 (Figure 2d). Splice site analysis predicted that a noncanonical 3′ acceptor site, 33 nucleotides downstream of the canonical exon 15 acceptor site, would be strengthened by the *TSC2* c.1477C>G substitution (Figure S2a). In the in vitro splicing assay, the *TSC2* c.1477C>G substitution resulted in utilization of this noncanonical site, leading to the production of an abnormal *TSC2* mRNA transcript, *TSC2* r.1444_1477delinsG, p.482_493delinsV (Figure 5). This was the major splice product: Sequence corresponding to the canonically spliced exon 14 was detectable, but was clearly the minor product in the presence of the *TSC2* c.1477C>G variant (Figure 5b).

The p.482_493delinsV variant (abbreviated to 482del11 in Figures 2 and 3) did not interact with TSC1 (Figure 3), and was unable to inhibit TORC1. The T389/S6K ratio was not significantly different from the T389/S6K ratio in the absence of TSC2 (Figure 2d). Therefore, although functional assessment did not strongly support the pathogenicity of the p.L493V amino acid substitution, our analysis indicates that the c.1477C>G nucleotide substitution is likely to inactivate *TSC2* due to a splicing defect, resulting in the production of inactive TSC2 p.482_493delinsV protein. Nonetheless, because we could not exclude the possibility that the canonically spliced *TSC2* c.1477C>G p.L493V variant is produced in vivo, we classified the *TSC2* c.1477C>G p.(L493V) variant in group 2 (Table 1).

The p.T147K, p.M280T, p.R308W, p.L369P, p.L448R, p.V504D, p.525del, p.S1045F, and p.I1197F variants were all associated with a significant reduction in the ability of the TSC complex to inhibit S6K‐T389 phosphorylation (*p* < .025; paired Student's *t* test vs. wild‐type TSC2, with Bonferroni correction). However, in each case, the variant retained some activity: The T389/S6K ratio was significantly reduced compared with cells not expressing TSC2 (*p* < .025; paired Student's *t* test, with Bonferroni correction; Figure 2b). The p.T147K, p.M280T, p.R308W, p.L369P, p.L448R, p.V504D, and p.525del variants were associated with reduced TSC1 signals (Figure 2c) and TSC complexes could not be coimmunoprecipitated efficiently (Figure 3), indicating that these amino acid changes affect the TSC1–TSC2 interaction. The T147K, M280T, and L369P substitutions are predicted to either cause a packing defect or disrupt helix formation of the TSC2 NT domain that is required for the interaction with TSC1 (Zech et al., 2016). The R308W substitution is not predicted to disrupt protein folding, but R308 is part of a surface loop that might directly bind TSC1 (Figure 4). Structural data on the region of TSC2 encompassing residues L448, V504, and N525 is not yet available, making predictions of the likely effects of these variants on the structure of the TSC complex difficult.

Functional assessment of the p.L369P, p.L448R, and p.525del variants was consistent with the clinical and genetic findings, and with the hypothesis that these variants cause TSC. The p.T147K, p.M280T, p.R308W, and p.V504D variants were less straightforward to interpret. The functional results suggested that these variants disrupt TSC complex activity. However, the individual with the p.T147K variant did not fulfill the clinical criteria for definite TSC (Northrup & Krueger, 2013), the p.M280T and p.V504D variants were identified in individuals without any clinical signs of TSC (see Table 2 and Figure S1) and the p.R308W variant has been reported in three individuals in the updated ExAC dataset (Genome Aggregation Database; MAFs of 0.005% and 0.002% in the East Asian and European populations, respectively).

In contrast to the p.T147K, p.M280T, p.R308W, p.L369P, p.L448R, p.V504D, and p.525del variants, the p.S1045F and p.I1197F substitutions did not significantly affect TSC1 signals (Figure 2d), and did not disrupt TSC complex formation (Figure 3). However, both variants affected the TSC complex function, resulting in significantly increased TORC1 activity (Figure 2d).

The *TSC2* c.3134C>T p.(S1045F) variant was identified in three affected individuals of a three generation family with TSC (Figure S1d). Although splice site analysis did not predict an effect of the c.3134C>T substitution on *TSC2* splicing (Figure S2d), an abnormal *TSC2* mRNA transcript lacking exon 28 and resulting in an in‐frame deletion of 50 amino acids (p.1044_1094del) was reported in an affected carrier (data not available) and confirmed in the in vitro splicing assay (Figure 5; Table 3). The resulting *TSC2* r.3132_3284del p.1044_1094del variant (abbreviated to 1044del50 in Figures 2 and 3) did not significantly affect TSC1 signals (Figure 2c) but completely prevented the TSC complex‐dependent inhibition of TORC1 (Figure 2d). Although the TSC2 p.1044_1094del variant was inactive (Group 3), we classified the *TSC2* c.3134C>T variant in Group 2 because we could not exclude the possibility that canonically spliced *TSC2* mRNA would still be expressed in vivo.

The p.I1197F variant was identified in an individual fulfilling the clinical criteria for TSC as well as in a parent without any clinical features of the disease. The I1197 residue is unlikely to be involved in the interaction with TSC1 (Zech et al., 2016) but maps close to a region that is important for TSC complex function (Wentink et al., 2012; Živčić‐Ćosić et al., 2017). Indeed, as estimated from the T389/S6K ratio (Figure 2d), TORC1 activity in the presence of the p.I1197F variant was increased approximately twofold relative to wild‐type TSC2. TSC1 signals (Figure 2c) and TSC complex formation (Figure 3) were not affected.

The *TSC2* c.4966G>T p.(D1656Y) substitution cosegregated with TSC in a 3 generation family. One individual was mosaic for the variant, and the clinical and genetic findings were strongly suggestive that the *TSC2* c.4966G>T p.(D1656Y) variant causes TSC (Table 2; Figure S1b). However, the functional assessment did not support the pathogenicity of either the p.D1656Y substitution or the p.D1656N substitution that is reported in a single individual in the gnomAD database (*TSC2* c.4966G>A p.(D1656N), rs773255614; population frequency <5 × 10^−6^ at https://gnomad.broadinstitute.org/). Both variants inhibited TORC1 activity as effectively as wild‐type TSC2 (Figure 2d) and neither affected the TSC1–TSC2 interaction (Figure 3). The D1656 residue is located within the TSC2 GAP domain (amino acids 1,538–1,729) but is most likely exposed on the exterior surface of TSC2, facing away from the RHEB binding site (Figure 4). It is therefore unlikely that either the D1656Y or D1656N substitution causes a major structural defect. Splice site analysis predicted that the *TSC2* c.4966G>T substitution, but not the *TSC2* c.4966G>A substitution strengthened a noncanonical 5′ donor site 29 base pair upstream of the canonical exon 38 donor site (Figure S2b,c). In the in vitro splice assay, the *TSC2* c.4966G>A substitution did not affect splicing: The canonical exon 38 splice product (*TSC2* nucleotides 4850_4989) was detected, similar to the wild‐type (Figure 5c). In contrast, the *TSC2* c.4966G>T substitution resulted in the utilization of the noncanonical donor site and premature truncation of the *TSC2* ORF (r.4961_4989del and p.E1655Pfs*41). However, because we could not exclude the possibility that canonically spliced *TSC2* mRNA encoding the p.D1656Y variant is expressed in vivo, we classified the variant in Group 2.

#### 3.2.3 Group 3 variants

The p.L916P and p.1570_1571del (abbreviated to 1570del2 in Figures 2 and 3) variants completely prevented the TSC complex‐dependent inhibition of TORC1 in our assay: The T389/S6K ratio in the presence of these variants was not significantly different from the T389/S6K ratio in the absence of TSC2 (*p* > .025; paired Student's *t* test vs. TSC1/S6K only, with Bonferroni correction; Figure 2d). Both variants map outside the TSC2 NT domain that is required for the interaction with TSC1 (Zech et al., 2016) and neither variant affected TSC1 signals (Figure 2c) or immunoprecipitation of the TSC complex (Figure 3). Structural information on the region encompassing the p.L916P variant is limited, making structural predictions difficult. The deletion of R1570 and Y1571 is predicted to disrupt the folding of the TSC2 GAP domain, consistent with the functional data (Figure 4).

## 4 DISCUSSION

**Figure 6:**
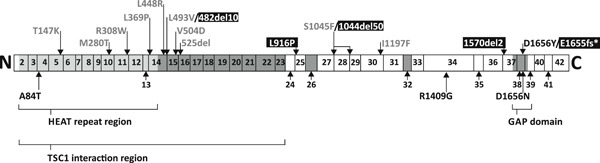
Schematic overview of the *TSC2* variants investigated as part of this study. The approximate positions of the variants, relative to the coding exons according to genomic reference sequence NG_005895.1, are indicated. Variants are colored according to their classification group, as shown in Figures 2 and 3: Group 1, black; Group 2, gray; Group 3, white (on black background); see main text for details. Alternatively spliced exons and regions of known structure and/or function are indicated with shading

We performed a functional assessment of 15 *TSC2* VUS identified in individuals suspected of TSC. In total, we characterized the effects of 18 different changes to the TSC2 protein. Our findings are summarized in Figure 6 and in Table 1, and have been submitted to the *TSC2* LOVD (http://www.lovd.nl/TSC2).

We identified four TSC2 variant isoforms that completely inactivated the TSC complex: p.482_493delinsV, p.L916P, p.1044_1094del, and p.1570_1571del. We consider the corresponding *TSC2* c.1477C>G, c.2747T>C, c.3134C>T, and c.4709_4714del variants as likely to be pathogenic, consistent with the clinical and genetic data.

TSC complex activity was significantly reduced by nine of the variants tested. In four cases, p.M280T, p.R308W, p.V504D, and p.I1197F the variant was identified in individuals without reported signs of TSC. It is possible that the differences we detected in our in vitro assays are not significant in vivo. However, no other candidate pathogenic *TSC1* or *TSC2* variants were identified in any of the affected individuals carrying the above variants. One possibility suggested by our functional data is that *TSC2* variants that do not completely inactivate the TSC complex might not be fully penetrant and therefore less likely to cause clinical TSC.

In three of our cases, p.A84T, p.R1409G, and p.D1656Y, we did not obtain evidence for an effect of the amino acid substitution on TSC complex function. The *TSC2* c.4966G>T p.(D1656Y) variant was identified in three affected individuals from a single family with TSC, one of whom was mosaic for the variant (Figure S1a) and the in vitro splice assay indicated that the variant affects *TSC2* pre‐mRNA splicing, resulting in premature truncation of the *TSC2* ORF. We did not obtain evidence for a splicing effect of either the *TSC2* c.250G>A p.(A84T) or c.4225C>G p.(R1409G) variants (Table 3).

For diseases like TSC that are associated with considerable phenotypic variation, it can be challenging to classify individuals based on the clinical signs only (Northrup & Krueger, 2013). We were able to distinguish variants that completely inactivated the TSC complex from those that had a partial effect and it would be interesting to establish whether these differences reflect differences in vivo. It is possible that we both under‐ and over‐estimated activity, depending on the particular variant being tested. We classified the variants based on comparisons to (a) wild‐type TSC2, (b) the inactive TSC2 p.R611Q variant, and (c) cells not expressing TSC2. However, it is not clear how much TSC complex activity is required to prevent TSC, and what the threshold TORC1 activity is, above which TSC lesions are likely to develop. For example, in the absence of TSC2, TORC1 activity (as estimated from the T389/S6K ratio) increased almost 10‐fold. However, in the presence of the p.R308W and p.I1197F variants, we detected only a twofold increase in TORC1 activity, compared with wild‐type TSC2. Although this represented a significant decrease in TSC complex activity, it is possible that both variants retain sufficient activity in vivo to restrain the TORC1 activity below a critical, pathological threshold.

So far, our functional data correlates well with the known structural features of the TSC complex. In the eight cases where comparisons were possible, the functional data were consistent with the structural predictions. More detailed insight into the three‐dimensional structure of the TSC complex and the exact nature of the intra‐ and intermolecular interactions that are critical for TSC complex integrity and activity might be one way to improve *TSC2* variant classification. In addition, more accurate assays of TSC complex activity would allow a more confident prediction of the likely pathogenicity of specific *TSC1* and *TSC2* variants. One shortcoming of our assay is that it relies on overexpression of the specific *TSC2* variants. It would be useful to compare *TSC1* and *TSC2* variants in their original genomic context and at endogenous expression levels. These approaches will be helpful because, as our study demonstrates, current functional tests are not always able to unequivocally establish or exclude pathogenicity. In two cases (13%), c.250G>A p.(A84T) and c.4225C>G p.(R1409G), the functional data supported the hypothesis that the variant did not cause TSC, but we could not exclude pathogenicity. In 13/15 cases (87%), the functional assessment supported the hypothesis that the identified variant was likely to cause TSC. However, in four cases, c.839T>C p.(M280T), c.922C>T p.(R308W), c.1511T>A p.(V504D), and c.3589A>T p.(I1197F), this was not consistent with all the clinical and genetic data. In three cases, c.1477C>G p.(L493V), c.3134C>T p.(S1045F), and c.4966G>T p.(D1656Y), analysis of *TSC2* pre‐mRNA splicing was essential for helping establish the likely pathogenicity of the variant. Our results demonstrate that the failure to show an effect on protein function is not sufficient to exclude pathogenicity and highlight some of the difficulties in classifying *TSC2* VUS and in assigning pathogenicity. It is clear that new functional assays that would enable a more accurate assessment of variant pathogenicity in TSC would be very useful. Nonetheless, despite the shortcomings highlighted above, our study confirms the utility of functional testing for *TSC2* VUS.

## CONFLICT OF INTERESTS

The authors declare that there are no conflict of interests.

## AUTHOR CONTRIBUTIONS

M. N., L. G. D. A., S. N., M. H., R. K.‐G., and P. E. performed the experiments. G. S. and A. K. provided assistance and materials for the generation of the CRISPR/Cas9 *TSC1* and *TSC2* knockout cells. D. K. performed the structural analysis. A. Z. and N. M. collected and analyzed the clinical and genetic data. R. E. and S. P. reviewed the genetic data and performed a critical revision of the manuscript for important intellectual content.

## Supporting information

Supporting information

## Data Availability

The data that support the findings of this study are available from the corresponding author upon reasonable request.
