# Supplementary material for: Comparison of the functional and structural characteristics of rare TSC2 variants with clinical and genetic findings
Source: Hum Mutat. 2019 Dec 19;41(4):759–73. doi: 10.1002/humu.23963 (PMC7154745; doi:10.1002/humu.23963)

**Supporting Information**

**Supp. Figure S1.** Pedigrees of families segregating the *TSC2* VUS tested as part of this study. Clear symbols indicate no signs or symptoms of TSC; filled symbols indicate individuals with, or suspected of, TSC (see Tables 1, 3 and 4 for details). Genotypes are shown for the individuals for whom DNA testing was performed; + indicates the presence of the *TSC2* VUS. ? indicates that clinical data was not available. (**A**) *TSC2* c.4966G>T (p.D1656Y); (**B**) *TSC2* c.839T>C (p.M280T); (**C**) *TSC2* c.1511T>A (p.V504D); (**D**) *TSC2* c.3134C>T (p.S1045F).


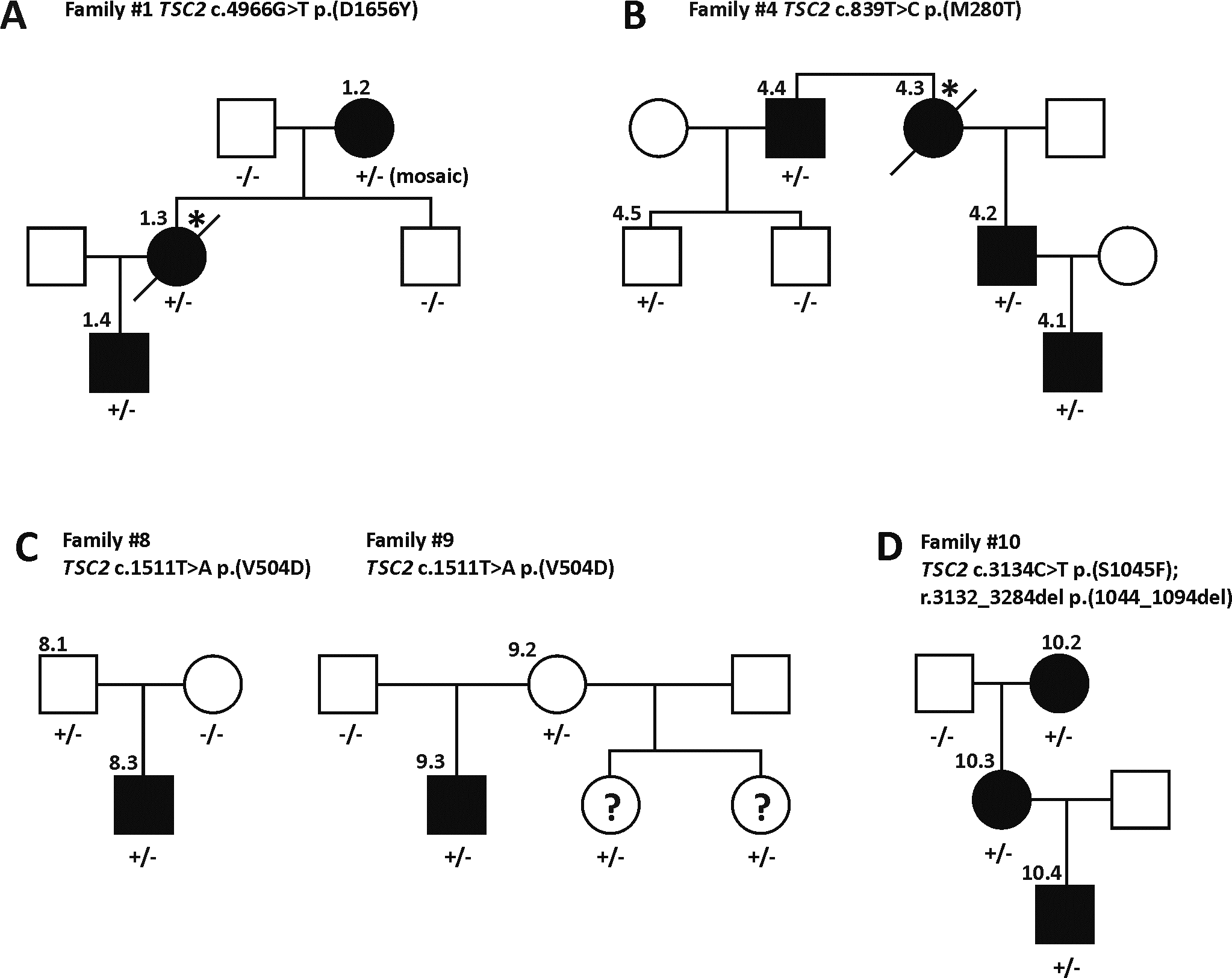


**Supp. Figure S2.** Splice site prediction analysis of the *TSC2* (NM_000548.3) c.1477C>G p.(L493V) (**A**), c.4966G>A p.(D1656N) (**B**), c.4966G>T p.(D1656Y) (**C**) and c.3134C>T p.(S1045F) variants (**D**).

**A**


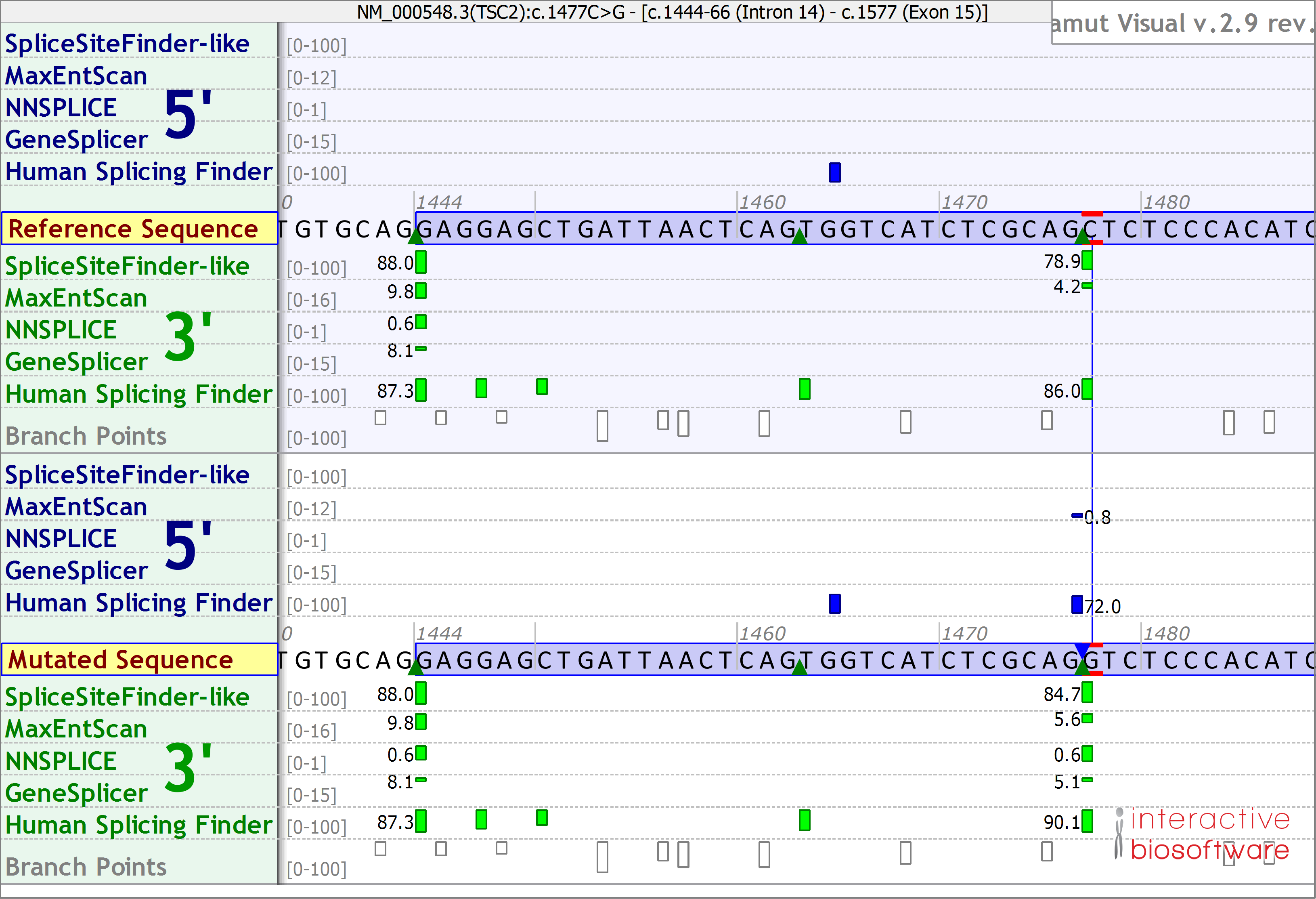


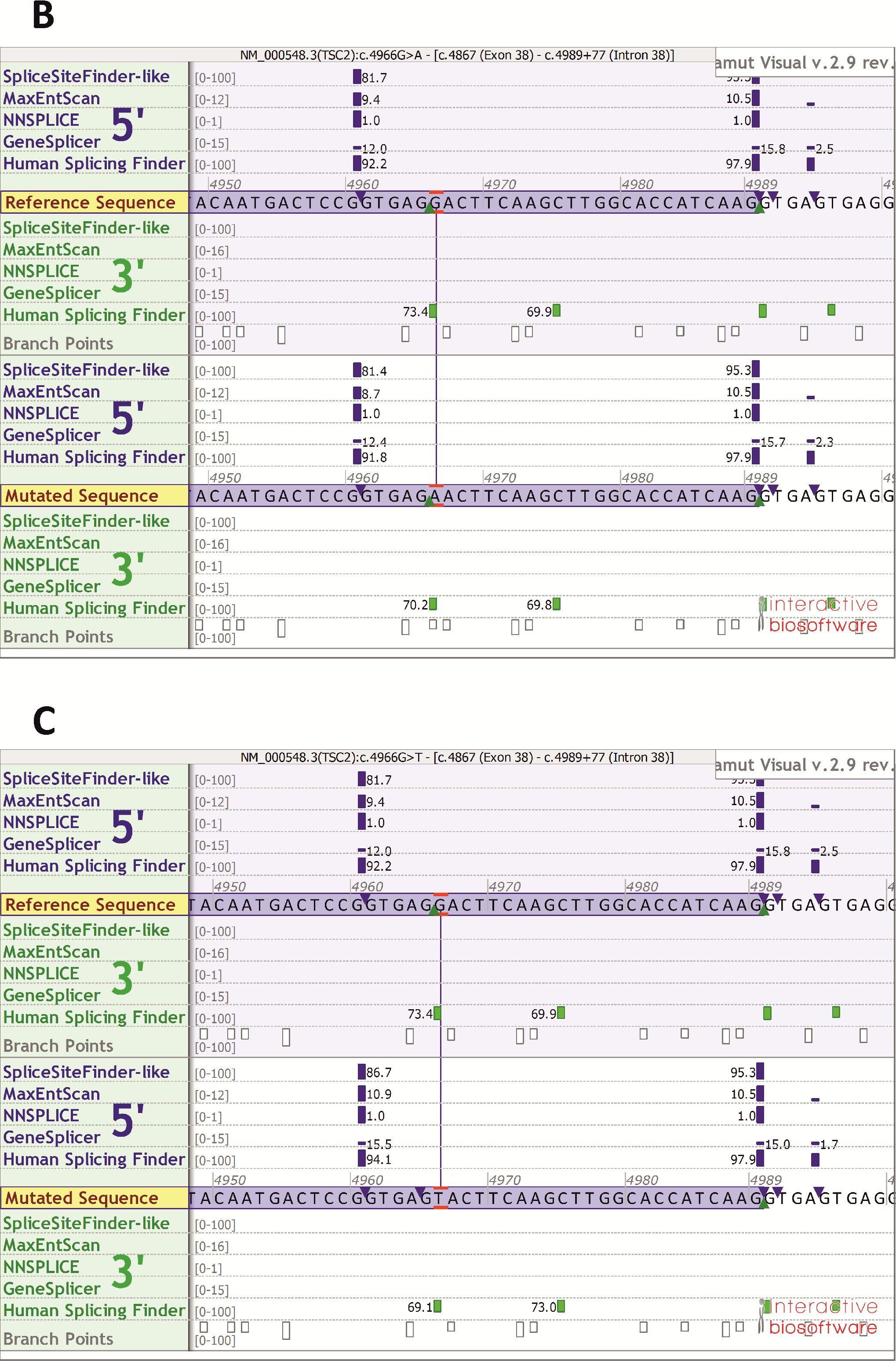


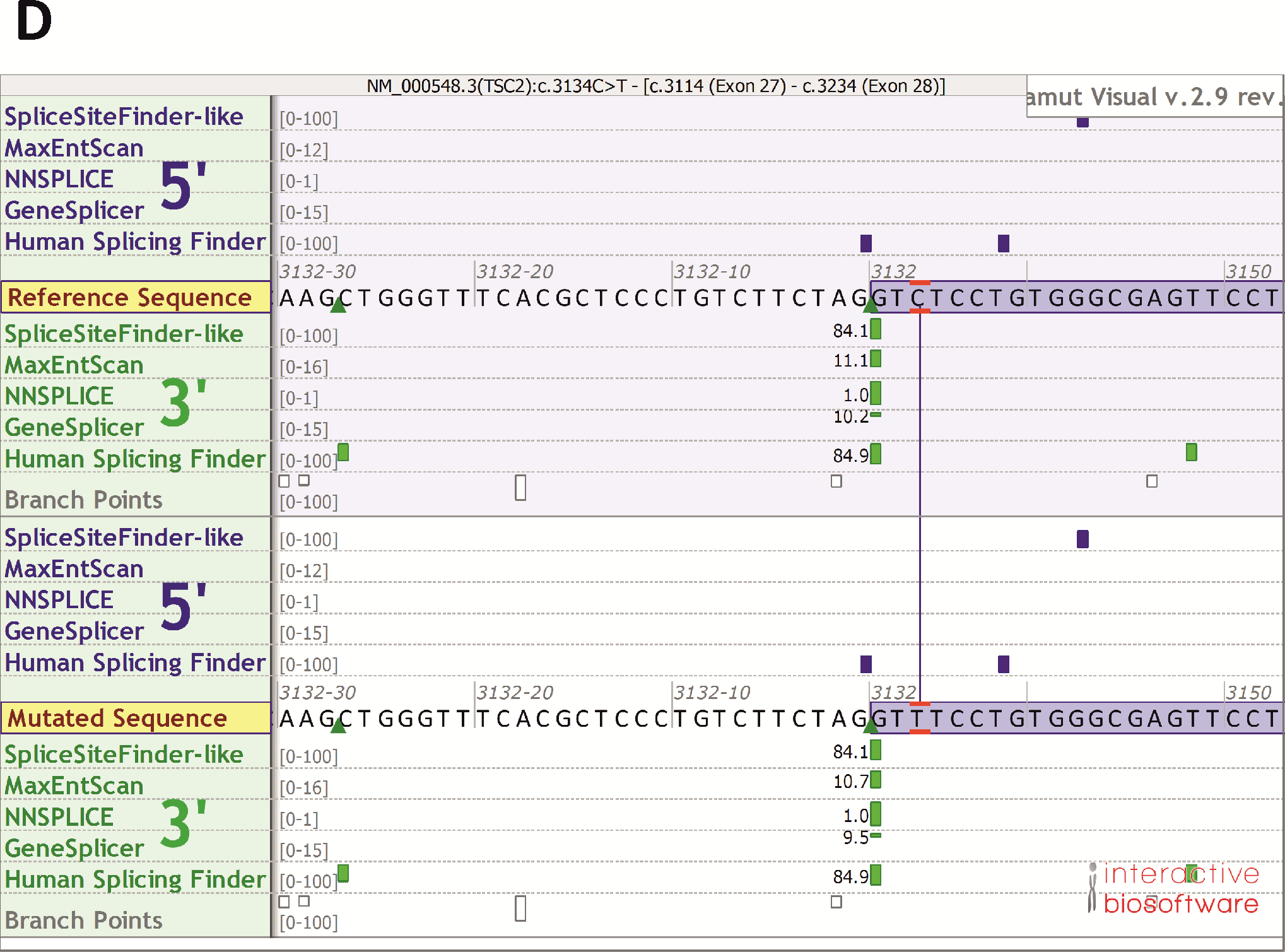

Supplement: Supplementary file 1 — Supporting information [file HUMU-41-759-s001.doc]
